# Simultaneous Determination of Columbianadin and Its Metabolite Columbianetin in Rat Plasma by LC-MS/MS: Application to Pharmacokinetics of Columbianadin after Oral Administration

**DOI:** 10.1155/2018/8568303

**Published:** 2018-09-19

**Authors:** Jin Li, Zhen Li, Qian Luo, Chun-Peng Wang, Jun He, Xiaoli Pang, John Teye Azietaku, Yan-xu Chang

**Affiliations:** ^1^Tianjin State Key Laboratory of Modern Chinese Medicine, Tianjin University of Traditional Chinese Medicine, Tianjin 300193, China; ^2^Tianjin Key Laboratory of Phytochemistry and Pharmaceutical Analysis, Tianjin University of Traditional Chinese Medicine, Tianjin 300193, China; ^3^Academy of Nursing, Tianjin University of Traditional Chinese Medicine, Tianjin 300193, China

## Abstract

Columbianadin and its metabolite columbianetin exhibited the anti-inflammatory, analgesic, calcium channel blocking and antitumor activities. To compare the differences between pharmacokinetics of columbianadin and its metabolite columbianetin after oral administration of pure columbianadin and* Angelicae Pubescentis Radix* (APR) extract, a simple and sensitive liquid chromatography-tandem mass spectrometry (LC-MS/MS) method was established and validated to simultaneously determine columbianadin and columbianetin in rat plasma. Two analytes and an internal standard (warfarin) were well separated and determined after liquid-liquid extraction with ethyl acetate. Ammonium acetate aqueous solution (1 mmol/L) and acetonitrile were used as the mobile phase and the flow rate was 0.3 mL/min. The lower limit of quantification (LLOQ) was 0.1 ng/mL for columbianetin and 0.5 ng/mL for columbianadin, respectively. There were significant differences between some pharmacokinetic parameters and bioavailability of columbianadin after oral administration of pure columbianadin and APR extract. The studies on comparative pharmacokinetics of columbianadin were of great use for facilitating the clinical application of columbianadin and were also highly meaningful for the potential development of APR.

## 1. Introduction

Herbal medicines were used for treatment of various diseases due to the synergistic or antagonistic actions of effective ingredients over thousands of years. The dynamic process of effective ingredients of herbal medicine* in vivo* was manifested by the pharmacokinetics. The pharmacokinetics study was helpful for investigating the efficacy and clinical applications of herbal medicine. Therefore, it is important to investigate the pharmacokinetics of the active ingredients in herbal medicine. The analysis of a single and pure ingredient may not represent the pharmacokinetics in whole herbal medicine due to the effect of other ingredients. In view of the above, it is necessary to develop a sensitive method for comparing the difference between pharmacokinetics of ingredient after oral administration of pure form and whole herbal medicine extract to rats.

*Angelicae Pubescentis Radix *(APR), known as Duhuo in Chinese, is one of well-known herbal medicines mainly used to treat rheumatism and headache in clinic according to Chinese Pharmacopoeia [1]. Columbianadin was one of the main active ingredients in APR extract [1, 2]. The previous studies have revealed that columbianadin possessed the hepatic-protective activity [3]. It was reported that columbianadin was converted into columbianetin* in vivo* [4]. Columbianetin was reported to have the antifungal activity [5]. In addition, columbianadin and columbianetin also exhibited the anti-inflammatory, analgesic, calcium channel blocking and antitumor activities [6–10]. Moreover, the intestinal absorption, transportation, and assessment of absorption characteristics have been investigated [11–13].

High performance liquid chromatography (HPLC) method has been applied to the pharmacokinetic study and tissue distribution of columbianadin [14, 15]. Columbianadin or columbianetin after an oral administration of pure ingredient or APR extract were also reported by LC-MS/MS in our previous researches [16–18]. Although these analytical methods in biological sample have been attempted, the method for simultaneous determination of columbianadin and its metabolite columbianetin has not been developed to evaluate the differences between the oral bioavailability of columbianadin and columbianetin after oral administration of pure columbianadin and APR extract. A sensitive and rapid LC-MS/MS method was described to simultaneously determine the concentrations of columbianadin and columbianetin in rat plasma in the present study. It was successfully applied to the oral bioavailability of columbianadin and pharmacokinetics of columbianetin after oral administration of pure columbianadin and APR extract. The oral pharmacokinetics of columbianadin and its metabolite were highly helpful to facilitate the reasonable application of APR in clinic and the further research of its major active component columbianadin.

## 2. Materials and Methods

### 2.1. Chemicals and Reagents

Columbianetin, columbianadin, and warfarin (internal standard, IS) were supplied from National Institute for the Control of Pharmaceutical and Biological Products (Beijing, China). HPLC grade of acetonitrile and methanol were obtained from Dikma technologies Inc (USA) and Tianjin concord Science Co. Ltd. (Tianjin, China), respectively. The analytical grade reagents were ethyl acetate and ammonium acetate. Deionized water was purified with a Milli-Q Academic ultra-pure water system (Millipore, Milford, MA, USA).

### 2.2. Liquid Chromatographic Conditions

The Agilent 1200 system (Agilent Corporation, USA) equipped with a vacuum degasser (G1322A), a binary pump (G1312A), and Hip-ALS autosampler (G13678). An Eclipse plus C_18_ (4.6 mm × 100 mm, 1.8 *μ*m) column with a security guard C_18_ column (2.1 mm × 12.5 mm, 5 *μ*m, Agilent, USA) kept at 25°C was used for the chromatographic separation. The mobile phases consisted of acetonitrile (A) and ammonium acetate aqueous solution (1 mmol/L) (B) with a gradient elution of 40% A at 0-5 min; 40%-70% A at 5-10 min; 70%-75% A at 10-11 min; 75%-90% A at 11-19 min; 90% A at 19-22 min; 90%-40% A at 22-29 min. The reequilibration time of gradient elution was set at 5 min. The flow rate was set at 0.3 mL/min and the injection volume was 15 *μ*L.

### 2.3. Mass Spectrometric Conditions

The detection was performed on an API 3200 triple quadrupole mass spectrometer with an electrospray ionization (ESI) source (Concord, Ontario, Canada). The multiple reaction monitoring (MRM) mode with the positive electrospray ionization was used for the quantitative transitions of* m/z* 247.3→ 175.0 for columbianetin, 329.3→ 229.3 for columbianadin and 309.2→ 163.2 for IS, respectively. The other MRM parameters were showed in Table 1. In addition, the optimum source parameters were as follows: curtain gas, 15 psi; collision gas, 5 psi; ion Spray voltage, 5500V; temperature, 350°C; ion source gas1, 40 psi; ion source gas2, 60 psi.

### 2.4. Preparation of Stock Solution, Calibration Samples, and Quality Control (QC) Samples

The accurately weighed analytes and IS were prepared in methanol at a concentration of 1.0 mg/mL as stock solutions. Stock solution of warfarin was diluted to 100 ng/mL as the final IS solution.

The appropriate standard solution of two analytes and 10 *μ*L IS was added to 100 *μ*L blank rat plasma to obtain a series of different concentrations of working solution samples as the calibration curve samples. Quality control (QC) samples were prepared at three levels (low, medium, and high concentrations).

### 2.5. Preparation of Sample

After thawing, the plasma samples (100 *μ*L) were spiked with 10 *μ*L IS solution. Ethyl acetate (1 mL) was added to extract two analytes. The mixture was swirled for 1 min and then centrifuged for 10 min at 14000 g. The supernatant was transferred to another centrifuge tube and then evaporated to dryness with nitrogen. The residue was reconstituted in 100 *μ*L methanol, followed by swirling for 1 min and centrifuging at 14000 g for 10 min. Finally, the supernatant (15 *μ*L) was used for analysis.

### 2.6. Method Validation

To validate the analytical method, the specificity, sensitivity, linearity, accuracy and precision, recovery and matrix effect, and stability were assessed in compliance with the USFDA guidelines.

#### 2.6.1. Specificity

The specificity was used to check whether endogenous substances interfered with analytes and IS or not. The specificity was evaluated by comparing the chromatograms of analytes-free plasma containing neither analytes nor IS (double blank) with those of corresponding spiked plasma and real plasma sample after administration.

#### 2.6.2. Linearity and Sensitivity

Calibration curves were investigated by determining the calibration curve samples at a series of different concentrations levels. The ratios of analyte to IS area versus analyte concentration were used for regression analysis. Each calibration curve was analyzed individually by using least square weighted (1/x^2^) linear regression. The lower limit of quantification (LLOQ), the lowest concentration of quantitative detection, could be measured with the relative standard deviation (RSD) (n = 6) within 20% and the signal-to-noise (S/N) ratio was less than 5.

#### 2.6.3. Accuracy and Precision

For assessing the intra- and interday accuracy and precision, replicate analysis of QC samples at three levels was performed on a single day and 3 consecutive days along with the standard calibration curve. The relative standard deviations (RSDs) were used to evaluate the intra- and interday variations. The RSD was expected to be within ± 15.0%. The accuracy was evaluated by the percentage of the calculated concentration to the nominal concentration.

#### 2.6.4. Recovery, Matrix Effect and Stability

The recoveries of columbianetin and columbianadin were tested by comparing their peak areas in extracted samples with those in postextracted spiked sample at three concentrations (low, medium, and high) with six determinations. The matrix effect was determined by comparing the peak areas in postextracted spiked samples with those of the analytes in unextracted samples at three concentrations.

The stability tests were designed to cover the anticipated conditions that the samples might be exposed during storage and handling, including reanalyzing QC samples at room temperature for 24 h, three freeze-thaw cycles, stored at - 20°C for 15 days. All stability studies were evaluated at three QC levels with six determinations for each test. For various conditions, QC samples were determined with the freshly prepared calibration curves.

### 2.7. Pharmacokinetic Study

Twenty male Sprague-Dawley (SD) rats (250-280 g) were kept in a special facility with food and water freely provided. The facility was lighted 12 hours per day and the room temperature was maintained at 22°C. After rats were fed for 7 days, they were fasted for 12 h with free access to water before the experiment. Twenty rats were randomly divided into two equal groups with oral administration of pure columbianadin at 25 mg/kg and APR extract at 3.74g/kg, respectively. The blood samples (approximately 250 *μ*L) were collected from their fossa orbitalis at 0.08, 0.25, 0.5, 0.75, 1, 2, 3, 4, 6, 8, 12, and 24 h. After blood was centrifuged at 4000 g for 10 min at 4°C, the plasma was harvested and stored at −20°C prior to analysis. The study was carried out after the approval from the independent animal ethics committee of Tianjin University of Traditional Chinese Medicine.

### 2.8. Data Analysis

According to DAS pharmacokinetic software package (version 1.0, Chinese Pharmacological Association, China, Anhui), the one-compartmental model was suitable to describe the pharmacokinetic parameters after oral administration. The main pharmacokinetic parameters such as the time to reach maximum drug concentration (T_max_) and the maximum plasma concentration (C_max_), half-life (t_1/2_), the area under the plasma concentration-time curve (AUC), and mean residence time (MRT) were calculated. The absolute bioavailability was calculated as follows: F= (AUC_(0-*∞*)oral_/AUC_(0-*∞*)intravenous_) × 100%, and AUC_(0-*∞*)intravenous_ was based on the result of columbianadin intravenous absorption in our previous paper [16].

## 3. Results and Discussion

### 3.1. Optimization of the Chromatographic Conditions and Sample Preparation

**Figure 1:**
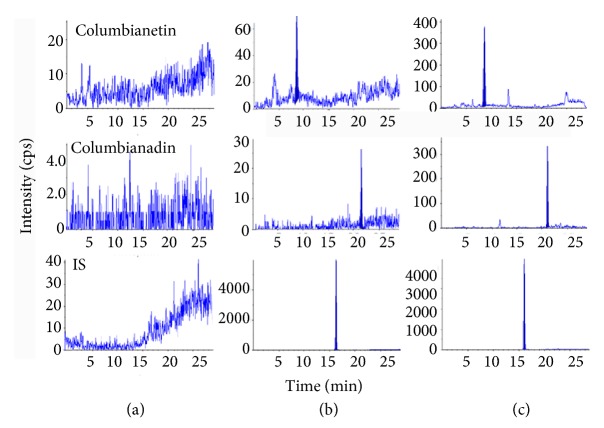
Representative MRM chromatograms: (a) blank plasma sample, (b) blank plasma sample spiked with columbianetin and columbianadin at LLOQ, and (c) real plasma sample after oral administrated of 25 mg/kg columbianadin.

In order to achieve the better resolution and sensitivity of all analytes, mobile phase, gradient elution type, flow rate, and type of column were optimized. Finally, the shorter run-time, best peak shape, and sensitive signal were obtained for two analytes and IS when Eclipse plus C_18_ column (100 mm × 4.6 mm, 1.8*μ*m) was used to separate all compounds. Ammonium acetate aqueous solution (1 mmol/L) and acetonitrile were selected as the mobile phase at a flow rate of 0.3 mL/min. According to previous research [16–18], ethyl acetate was employed for sample preparation. It was found that ethyl acetate provided a much cleaner supernatant and a satisfactory and stable recovery. As shown in Figure 1, interfering peaks in their peak region by analyzing six different blank plasma samples did not be discovered.

### 3.2. Selection of Internal Standard

To determine the concentration of columbianetin and columbianadin in rat plasma after an oral administration of 25 mg/kg columbianadin, warfarin was selected as IS based on the similar chemical structures, chromatographic performance, and ionization under the same analytical conditions.

### 3.3. Assaying the Dose of Oral Administration of APR Extract

The contents of columbianetin and columbianadin in APR extract were determined by LC-MS/MS. The results showed that the contents of columbianetin and columbianadin in APR extract were 0.11 mg/g and 6.68 mg/g, respectively. Based on the dose of columbianadin of 25 mg/kg, the calculated dose of the APR extract is 3.74 g/kg, equivalent to oral administration of the same dose of columbianadin.

### 3.4. Method Validation

As shown in Figure 1, the analytes and IS were well separated and no endogenous substances or metabolites interfered with two analytes at the same retention time. The good linearity of columbianetin and columbianadin (Table 2) was achieved when the calibration curves were established by the peak area ratio of analytes to IS (Y) versus analyte concentration (X) over the linear concentration ranges and the weighing factor was 1/x^2^. The LLOQ of columbianetin and columbianadin in rat plasma was 0.1 ng/mL and 0.5 ng/mL, respectively. The data of accuracy and precision of intra- and interday are listed in Table 3. The results indicated that the method was accurate, reliable, and precise. The extraction recoveries which were evaluated by determining QC samples (n = 6) at three levels are also summarized in Table 3. It could be seen that the mean extraction recoveries (low, medium, and high concentrations) ranged from 81 to 102% with RSDs of 6-10%. Meanwhile, the extraction recovery of IS also meet the analytical requirement. The matrix effects of columbianetin and columbianadin (Table 3) were within 85.6-99.7% with RSDs less than 12% and the matrix effects of IS were also within the acceptable limits, which indicated that no significant interference complicated the determination of analytes as well as IS. Thus, the ion suppression or enhancement from rat plasma matrix could be negligible. Additionally, these results of extraction recoveries and matrix effects also demonstrated that the method of liquid-liquid extraction with ethyl acetate was efficient and acceptable. The stability of columbianetin and columbianadin in rat plasma at 4°C (Table 3) in the autosampler for 24 h and three freeze-thaw cycles at −20°C for at least for 15 days was evaluated. It was observed that the concentration stability of columbianetin and columbianadin was within 97.0-102% of their nominal values and the significant degradation was not found, which demonstrated two analytes in rat plasma were stable under those conditions. All above results proved that the newly developed LC-MS/MS method was sensitive, reliable, and enough for the simultaneous determination of columbianetin and columbianadin in rat plasma.

### 3.5. Pharmacokinetic Study

#### 3.5.1. Selection of Compartment Model

**Figure 2:**
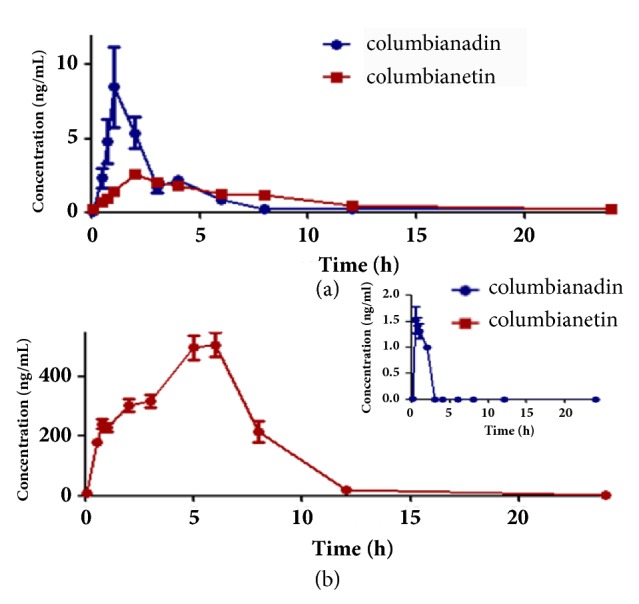
The mean plasma concentration-time profiles of columbianadin and columbianetin after oral administration: (a) columbianadin and (b) APR extract (n = 10, mean ± SD).

The validated LC-MS/MS method by which the concentration of columbianadin and columbianetin were simultaneously monitored was successfully applied to the pharmacokinetic studies after oral administration of pure columbianadin and APR extract to rats. The mean plasma concentration-time profiles of columbianetin and columbianadin are illustrated in Figure 2. The corresponding pharmacokinetic parameters are shown in Table 4. According to AIC comparison and model diagnostics, the one-compartment model provided the best fit for the plasma concentration-time data, due to the lowest Akaike's information criterion (AIC) value under equal weight scheme.

#### 3.5.2. Comparative Pharmacokinetics of Columbianadin after Oral Administration of Pure Columbianadin and ARP Extract

It could be found that columbianadin was quickly absorbed into the blood after oral administration of pure columbianadin and ARP extract (Figure 2). After oral administration of pure columbianadin, T_max_ was 3.03 ± 1.87 h which was significantly greater than 0.55 ± 0.33 h following oral administration of APR extract. This observation suggested that columbianadin of APR extract group could reached the maximum plasma concentration more quickly than pure columbianadin group. It was obviously that APR extract group had a relatively low maximum plasma concentration and less abundant exposure* in vivo* (C_max_ of 1.82 ± 0.64 ng/mL and AUC_(0-tn)_ of 1.05 ± 1.02 ng/mL h) compared with C_max_ of 13.33 ± 25.37 ng/mL and AUC_(0-tn)_ of 28.80 ± 41.46 ng/mL h of pure columbianadin group. In addition, the elimination rate of columbianadin of APR extract group (t_1/2_ of 0.92 ± 0.36 h) was obviously rapider than that of pure columbianadin group (t_1/2_ of 5.15 ± 4.58 h). Based on the different pharmacokinetic characteristics of pure columbianadin and APR extract group, it can be concluded that other components of APR extract promoted metabolism of columbianadin. These different pharmacokinetic results also indicated that oral administration of pure columbianadin may be more beneficial for the clinical efficacy of columbianadin.

#### 3.5.3. Comparative Oral Bioavailability of Columbianadin after Oral Administration of Pure Columbianadin and ARP Extract

After oral administration of APR extract, oral bioavailability of columbianadin was 0.016 ± 0.016% which was less than 0.44 ± 0.63% following administration of pure columbianadin. These results suggested that oral bioavailability of columbianadin might be reduced by other ingredients in APR extract. It can be concluded that oral administration of pure columbianadin, which possessed a relatively high bioavailability, may be more effective than oral administration of APR extract in clinic.

#### 3.5.4. Pharmacokinetics of Columbianetin

As depicted in Figure 2, columbianetin was quickly monitored after oral administration of pure columbianadin. This result indicated that columbianadin could be transformed into columbianetin* in vivo*, which was consistent with the previous report [4]. After oral administration of pure columbianadin and APR extract, the maximum plasma concentrations of columbianetin (C_max_ of 3.12 ± 0.93 ng/mL and 631.40 ± 427.10 ng/mL) were achieved at 3.44 ± 1.95 h and 4.60 ± 1.65 h, respectively. The value of t_1/2_ was 3.44 ± 1.95 h and 4.52 ± 3.19 h for pure columbianadin and APR extract group, which indicated that the elimination of metabolite columbianetin was slow* in vivo*. After oral administration of pure columbianadin, approximately 0.12% columbianadin was transformed into columbianetin. The low conversion rate indicated that columbianetin was not a major metabolite of columbianadin* in vivo*.

## 4. Conclusion

A sensitive and rapid LC-MS/MS method was established to simultaneously determine columbianadin and its metabolite columbianetin in rat plasma. This LC-MS/MS method has been successfully applied for comparative pharmacokinetics of columbianadin and columbianetin after oral administration of pure columbianadin and APR extract to rats. The significant differences in pharmacokinetics and bioavailability of columbianadin were found between the pure columbianadin and APR extract group. The result indicated that oral administration of pure columbianadin may be more beneficial for the clinical efficacy of columbianadin. The studies on comparative pharmacokinetics, oral bioavailability, and metabolite were highly meaningful for the potential development and clinical application of APR.

## Figures and Tables

**Table 1:** MRM parameters of the three compounds.

Compounds	Q1	Q3	DP(V)	EP(V)	CE(V)	CXP (V)
Columbianetin	247.3	175.0	60	6	31	14
Columbianadin	329.3	229.3	81	3	13	20
Warfarin	309.2	163.1	60	7	20	13

DP: declustering potential; EP: entrance potential; CE: collision energy; CXP: collision cell exit potential.

**Table 2:** The calibration curves, linearity range, and LLOQs of the assay (n=6).

Compounds	Regression equation	r	Linearity range (ng/mL)	LLOQ (ng/mL)	Relative error (%)	RSD (%)
Columbianetin	Y=0.0412X+0.00654	0.9988	0.1-20	0.1	104	9.8
Columbianadin	Y=0.00431X+0.00164	0.9966	0.5-20	0.5	101	9.0

**Table 3:** Recoveries, matrix effects, stability and intraday and interday accuracy, and precision of the columbianetin and columbianadin (n = 6).

Compound	Concentration (ng/mL)	Recovery		Matrix effect		Freeze-thaw three cycles		-20°C for 2 weeks		Autosampler for 24 h		Intraday		Interday	
		Mean (%)	RSD (%)	Mean (%)	RSD (%)	Remains (%)	RSD (%)	Remains (%)	RSD (%)	Remains (%)	RSD (%)	Accuracy (%)	RSD (%)	Accuracy (%)	RSD (%)

Columbianetin	0.5	89.2 89.7 88.2	2.03	96.0 93.1 95.6	2.12	105	5.51	112	5.72	96.8	1.82	101	2.61	96.4	7.02
	4 20		2.43 4.83		3.53 5.52	101 95	1.87 4.36	107 93.8	5.15 5.79	88.7 88.8	4.6 2.88	98.3 94.9	2.78 11.7	97.9 97.4	10.0 10.9
Columbianadin	0.5	92.8	2.88	93.8	4.18	99.4	2.13	98.6	2.27	102	6.72	94.1	1.95	98.7	4.08
	4	94.2	1.79	90.7	4.05	106	5.01	101	2.66	101	0.8	98.3	3.63	100	2.79
	20	95.9	5.44	96.1	10.0	93.2	6.21	101	4.63	106	6.05	94.4	6.95	99.2	7.03

**Table 4:** Main pharmacokinetic parameters of columbianadin and columbianetin in rats after oral administration (n =10, mean ± SD).

	Administration of columbianadin		Administration of APR extract	
Parameters	Columbianadin	Columbianetin	Columbianadin	Columbianetin
t_1/2_(h)	5.15 ± 4.58	3.44 ± 1.95	0.92 ± 0.36	4.52 ± 3.19
T_max_(h)	3.03 ± 1.87	3.30 ± 1.89	0.55 ± 0.33	4.60 ± 1.65
*C*_max_ (ng/mL)	13.33 ± 25.37	3.12 ± 0.93	1.82 ± 0.64	631.40 ± 427.10
AUC_(0-tn)_(ng/mL h)	28.80 ± 41.46	18.51 ± 13.26	1.05 ± 1.02	3346 ± 2550
AUC_(0-*∞*)_ (ng/mL h)	47.95 ± 66.80	23.88 ± 15.53	3.23 ± 1.46	3490 ± 2489
MRT_(0-tn)_(h)	6.38 ± 4.96	5.76 ± 1.65	0.78 ± 0.20	4.79 ± 0.76
MRT_(0-*∞*)_(h)	6.37 ± 1.46	8.72 ± 2.14	1.99 ± 0.59	5.67 ± 1.11

## Data Availability

The data used to support the findings of this study are included in this article.
